# Impact of different plant growth promoters on the physicochemical properties, volatile compounds, and antioxidant capacity of *Allium ramosum* flowers

**DOI:** 10.3389/fpls.2025.1606438

**Published:** 2025-10-06

**Authors:** Rui Xu, Mengli Qiu, Youchuan Chang, Ziyuan Yan, Zichan Li, Xu Han, Gang Hu, Guixia Liu

**Affiliations:** ^1^ School of Life Sciences, Hebei University, Baoding, China; ^2^ Fishers Mountain Pasture, Yuer Mountain Town, Chengde, Hebei, China; ^3^ The Engineering Research Center of Ecological Safety and Conservation in Beijing-Tianjin-Hebei (Xiong’an New Area) of MOE, Baoding, Hebei, China

**Keywords:** amino acid fertilizer, algal extract, antioxidant activity, quality, volatile compounds

## Abstract

Plant growth promoters regulate the production of bioactive compounds within plants, stimulating the accumulation of aromatic substances. However, the potential mechanisms by which amino acid fertilizers (Feihong Fertilizer Co., Ltd., main components: organic matter 13.9%, total nitrogen 2.3%, total phosphorus anhydride 0.2%, total potassium oxide 0.3%, chlorine 0.1%, sodium less than 0.1%) and algal extracts (Hangzhou Qingyang Technology Co., Ltd., main components: calcium+magnesium ≥100g/L, marine minerals ≥ 5%), influence the characteristic flavor compounds and antioxidant activity of *Allium ramosum* flowers remain unclear. Amino acid fertilizers and algal extracts were applied in combination with different substrates to investigate their effects on the dry weight, fresh weight, inflorescence stem height, corolla diameter, total ascorbic acid content, glutathione content (GSH), proline content, polyphenol content, flavonoids content, flavones content, soluble protein content, soluble sugars content, antioxidant activity, and volatile compounds of *Allium ramosum* flowers. We found that amino acid fertilizers and algal extracts in combination with compound microbial fertilizers significantly increased the content of polyphenols and flavonoids (The increases were 56.1% and 57.1%, respectively), total ascorbic acid content and glutathione content (GSH)(The increases were 154.5% and 58.2%, respectively), and reduced the content of malondialdehyde (MDA) and superoxide anion (O2• −). Additionally, these treatments significantly improved the 2,2-diphenyl-1-picrylhydrazyl radical scavenging rate, ABTS scavenging rate, iron reducing antioxidant power, superoxide anion radical scavenging rate (SASR), hydroxyl radical scavenging activity (HRSC), POD, SOD, and PPO activity(The increases were 11.6%, 63.8%, 173%, 105%, 53%, 56.1%, 56.2%, and 71.8%, respectively), and increased the antioxidant activity and volatile compound content of *Allium ramosum* flowers, thereby improving their postharvest quality and shelf life. In summary, the application of amino acid fertilizers and algal extracts had a positive effect on the growth, quality, antioxidant activity, and flavor of *Allium ramosum* flowers, thereby increasing their commercial value.

## 1 Introduction

The genus Allium encompasses hundreds of species and is an important edible plant used in traditional and modern medicine for its therapeutic properties, including antimicrobial, lipid-lowering, cardiovascular-protective, cholesterol-lowering, antithrombotic, hypoglycemic, and antitumor activities (Abdelrahman et al., 2020). The development of these diseases is often triggered by oxidative stress (Abdelrahman et al., 2020). Allium plants are known for their high content of flavonoids, organosulfur compounds, and phenolic compounds, which contribute to their strong antioxidant properties (Melguizo-Rodríguez et al., 2022; Cakmakci et al., 2022). Pradeep and Srinivasan (2017) found that onions rich in organosulfur compounds (la cipolla) and fenugreek seeds (*Trigonella foenum-graecum*) have beneficial effects on hyperglycemia and related metabolic disorders. Additionally, Allium plants can produce sulfur-containing metabolites, such as S-alk(en)yl-L-cysteine sulfoxides (CSOs), which have significant nutritional and medicinal value (Smith, 2003). CSOs are the primary flavor precursors in Allium vegetables (Liu et al., 2021; Maciel et al., 2021). The flavor of Allium plants is produced when cell rupture occurs and the enzyme alliinase hydrolyzes CSOs, resulting in the production of pyruvic acid, which is comparable to CSOs in the hydrolysis reaction (Wall and Corgan, 1992; Rattanachaikunsopon and Phumkhachorn, 2008; Hong et al., 2014; Zeng et al., 2017; Ning et al., 2023). Additionally, Allium plants are considered a health food that can improve kidney function and are used in traditional Chinese medicine to enhance sexual function, nocturnal diarrhea, abdominal pain, and diarrhea. *Allium ramosum*, a perennial herb of the Allium genus in the lily family, has gained attention for its unique flavor and rich nutritional value. The flowers of *Allium ramosum*, as the reproductive organ of the plant, possess a distinctive flavor and are rich in a variety of bioactive compounds, making them a highly potential edible and medicinal resource. Studies have shown that the flowers of *Allium ramosum* are rich in carotenoids, proteins, starch, and other nutrients, with their content even surpassing that of *Allium ramosum* leaves, making them high-quality food material (Zhen et al., 206). Therefore, enhancing the nutritional quality, antioxidant activity, and volatile compounds of *Allium ramosum* flowers may be a good strategy to improve the potential health benefits of Allium vegetables, including *Allium ramosum*.

Algal extracts have been extensively studied as plant growth stimulants (Kapoore et al., 2021; Michalak et al., 2016; Valverde et al., 2022). The influence of the algal extracts on plant photosynthesis, growth, quality, antioxidant potential, and volatile compound content has previously been validated (Senousy et al., 2023; Elakbawy et al., 2022; Suresh et al., 2019; Ahanger et al., 2019), with studies showing that algal extracts can enhance plant photosynthesis, promote chlorophyll synthesis and accumulation, and improve photosynthetic efficiency (Senousy et al., 2023; Suresh et al., 2019). Algal extracts have been shown to promote plant growth by increasing the stem length, leaf number, and root development in tomato seedlings (Elakbawy et al., 2022). Research has also shown that the plant hormones in algal extracts, such as cytokinins and auxins, can promote plant growth and development, increasing yield and quality (Bano et al., 2022). Furthermore, algal extracts can enhance plant antioxidant capacity by increasing the activities of antioxidant enzymes (peroxidase (POD), superoxide dismutase (SOD), and catalase (CAT)); while enhancing the proline and flavonoid content; scavenging reactive oxygen species (ROS); reducing oxidative damage; and improving the tolerance of plants to oxidative stress (Ahanger et al., 2019; Huseynova, 2012; Zheng et al., 2016; Sun et al., 2016). In recent years, the application of amino acid fertilizers as foliar fertilizers has garnered increasing attention, demonstrating significant advantages in the enhancement of plant quality, growth, and antioxidant capacity (Zhang et al., 2022; Kapoore et al., 2021; Melguizo-Rodríguez et al., 2022). Amino acid fertilizers, characterized by an abundance of various amino acids, serve as organic nutrients that are readily assimilated and employed by plants to enhance their growth and increase yield (Tóth et al., 2022; Brankov et al., 2020). Studies have shown that amino acid fertilizers can increase plant height, stem diameter, fruit size, fruit number, and weight; while improving fruit sugar content, vitamin C content, and other quality indicators (Li et al., 2024). Amino acid fertilizers not only promote plant growth, but also enhance the antioxidant capacity of plants, strengthening their tolerance to adverse conditions such as drought and salinity. Studies have also indicated that amino acid fertilizers can induce the activity of antioxidant enzymes (POD and CAT) in plants, reducing the damage that can result from environmental adversity (Zhang et al., 2022; Tóth et al., 2022; Huang et al., 2023). Furthermore, amino acid fertilizer treatment has been found to increase the SOD, proline, and flavonoid content of olive fruits, enhancing the stress tolerance of the plants (Haghighi et al., 2022; Li et al., 2023). These results suggest that amino acid fertilizers not only improve plant nutritional quality but also enhance their antioxidant capacity and disease resistance (Haghighi et al., 2022).

In summary, amino acid fertilizers and algal extracts (The main component is *phaeophyceae*), as novel plant biostimulants, have the potential to promote plant growth, increase yield and quality, and enhance stress resistance, offering broad application prospects in the development of sustainable agriculture. However, there are few reports on the effects of exogenous application of amino acid fertilizers or algal extracts on the growth, antioxidant capacity, and volatile substances of *Allium ramosum*. Therefore, this study investigates the effects of combined microbial fertilizers with amino acid fertilizers, combined microbial fertilizers with algal extracts, chemical fertilizers with amino acid fertilizers, and chemical fertilizers with algal extracts on the growth, antioxidant capacity, quality, and volatile compounds of *Allium ramosum* flowers(These combinations were chosen based on the characteristics of the fertilizers and the growth requirements of *Allium ramosum*. Microbial fertilizers are known to improve soil health and provide slow-release nutrients, while chemical fertilizers offer immediate nutrient availability. Amino acid fertilizers and algal extracts are expected to enhance nutrient uptake and stress tolerance. By combining these fertilizers, we aim to create a balanced nutrient supply that supports optimal growth, quality, and stress resistance in *Allium ramosum*.). The aim is to determine the suitable schemes to optimize cultivation outcomes of *Allium ramosum* flowers.

## 2 Materials and methods

### 2.1 Materials and cultivation treatments

*Allium ramosum* was transplanted in Yu’er Mountain Pasture, Chengde City, Hebei Province, and the experiment was conducted from May to August 2023. The study field is located 41°44’ N and 140°16’ E at an altitude of 1460 m. The pasture is situated in a semi-arid continental monsoon climate zone with an accumulated temperature of 1513.1°C when the temperature is ≥10°C and a frost-free period of 85 days. The experiment was conducted using a completely randomized block design with a plot size of 4 x 9 = 36 m^2^ and a 0.5 m interval between blocks. Two types of base fertilizers were used: conventional chemical fertilizer (The nitrogen fertilizer is urea containing 47% N, the phosphorus fertilizer is superphosphate containing 12% P_2_O_5_, and the potassium fertilizer is potassium chloride containing 50% K_2_O.) and 225.00 kg/hm^2^ compound microbial fertilizer (The compound microbial fertilizer is “Kunyijian Huolin Potassium Compound Microbial Fertilizer” manufactured by Tianjin Kunhe Biotechnology Group Co., Ltd. The nutrient content is as follows: N 3.76%, P_2_O_5_ 9.41%, K_2_O 2.83%, and the number of effective living bacteria ≥ 2.0 billion/ml.). The foliar fertilizers included 0.2% amino acid solution and 0.2% algal extract (After applying the base fertilizer, spray every ten days, for a total of three times.). The following experimental treatments were used: CK (control: No treatment was applied), C+A (conventional chemical fertilizer combined with 0.2% amino acid solution), C+S (conventional chemical fertilizer combined with 0.2% algal extract), B+A (225.00 kg/hm^2^ compound microbial fertilizer combined with 0.2% amino acid solution), and B+S (225.00 kg/hm^2^ compound microbial fertilizer combined with 0.2% algal extract).

### 2.2 Sample collection and preparation

For statistical analysis, one square meter was randomly selected from each block and some samples were analyzed for physiological indicators at −80°C, while the rest were blanched at 105°C for 10 min and then dried to constant weight at 75°C for further experimentation.

### 2.3 Growth parameters

To measure growth parameters, In each experimental plot, a one-square-meter area located within the interior of the plot is randomly selected as the sampling point and inflorescence stem height was measured using a ruler and corolla diameter using a Vernier caliper (victor vc5150s made in China). The fresh weight (FW) and dry weight (DW) of the *A. ramosum* flowers were measured using an electronic scale (made in China).

### 2.4 Antioxidant capacity

The scavenging activity of 2,2-diphenyl-1-picrylhydrazyl (DPPH) against free radicals was assessed following the protocol outlined by Xue et al. (2021) with the findings presented as the percentage of the dry weight (% DW). The iron-reducing antioxidant capacity of the *A. ramosum* flowers was measured using a ferric reducing antioxidant power (FRAP) kit according to with the manufacturer’s instructions (Beijing Solabao, Beijing, China). Briefly, flower tissue (0.1 g) was ground in 1 ml of pre-cooled extract, the homogenate centrifuged at 4°C for 10 min at 10,000×g, and the supernatant collected for detection.

The respective solutions were then combined according to the instructions provided with the FRAP kit and allowed to react in the dark at room temperature for 20 min. The absorbance at 593 nm was quantified using a spectrophotometer (UV-1100 spectrophotometer, Shanghai Mepu Instruments Co., Ltd, China), and the antioxidant potential of *A. ramosum* flowers was quantified by monitoring the change in absorbance, using Trolox as a standard control. The efficacy of 2,2’-Azino-bis(3-ethylbenzothiazoline-6-sulfonic acid) diammonium salt (ABTS) in neutralizing free radicals was assessed using the method outlined by Schaich et al. (2015), and the results reported as percentage of dry weight (% DW). The hydroxyl radical scavenging capacity (HRSC) was measured using the method described by Schaich et al. (2015), and the results were reported as a percentage. The superoxide anion radical scavenging rate was quantified using the same method, and the results were expressed as percentages.

### 2.5 Measurement of antioxidant enzyme activity

The SOD activity was quantified using the method described by Hosseinpour et al. (2020), and the results were reported as units per gram of FW per minute (U g^–1^ min^–1^ FW). The POD activity was measured following the protocol outlined by Khan et al. (2022) and the results were expressed as units per gram of FW per minute (U g^–1^ min^–1^ FW). Polyphenol oxidase (PPO) activity was assessed using the method described by Rasmussen et al. (2021) and the results were reported as units per gram FW per hour (U g^–1^ h^–1^ FW).

### 2.6 Determination of ROS and membrane lipid peroxidation

Methodology by Kamran et al. (2021) was employed to ascertain the rate of superoxide anion (O2• −) production, and concentration of malondialdehyde MDA (The product has a maximum absorption peak at 532nm and a minimum absorption peak at 600nm.) in *A. ramosum* flowers, with the results documented in moles per gram of FW [mol g^−1^ (FW)], millimoles per gram FW per minute [mmolg^−1^min^−1^ (FW)], and nanomoles per gram of FW (nmol g^−1^ (FW), respectively.

### 2.7 Nutritional parameter analysis

The GSH content was quantified using the method described by Zhong et al. (2023), with results reported as milligrams per gram of FW (mg g^–1^ (FW)). Free amino acid content was measured using the ninhydrin colorimetric method outlined by Xu et al. (2022), and the results expressed as milligrams per gram of DW (mg g^–1^ (DW)). The soluble sugars (SS) content was determined using the method reported by Kumar and Kumar (2023). The soluble protein (SP) content was measured using the method described by Xu et al. (2022). The total ascorbic acid (TAA) content was quantified according to the method described by Bida-Badi et al. (2023). Proline content was determined using the method described by Ghafoor et al. (2020), the flavonoid content was measured using the method described by Guo et al. (2022), and polyphenols were determined using the method described by Yang et al. (2018).

### 2.8 HS-GC-MS

Precisely weight 3g of *Allium ramosum* flower (freshly harvested samples) samples and place them in a 20mL headspace vial, which is then immediately sealed for metabolomics analysis using headspace-coupled gas chromatography-mass spectrometry (HC-GC-MS). The samples are injected into the GC-MS system using split mode, with an injection volume of 1 µL and a split ratio of 10:1. After separation through a VF-WAXms capillary column (25m×0.25mm×0.2µm, Agilent CP9204), the samples are subjected to mass spectrometry detection. The inlet temperature is set at 180°C, with high-purity helium gas as the carrier gas at a flow rate of 2 mL/min, and the septum purge flow rate is 3 mL/min. The temperature program starts at 40°C, holds for 2 minutes, then increases to 100°C at a rate of 5°C/min, followed by a further increase to 230°C at a rate of 15°C/min, and holds for 5 minutes, with a post-run at 230°C for 2 minutes. The electron impact ion source (EI) is used, with a transfer line temperature of 310°C, an ion source temperature of 230°C, a quadrupole temperature of 150°C, and an electron energy of 70 eV. The scanning mode is full scan (SCAN), with a mass scan range of m/z 30-1000, and a scan frequency of 3.2 scans/s.

### 2.9 Quality control

To evaluate the stability of the analysis system during the testing process, a quality control sample (QC: Mix all the samples to be tested according to the same mass.) is prepared. The QC sample is a mixture of all the samples to be tested and is treated in the same manner as the formal samples. During the instrument analysis, a QC sample is inserted every 5-15 samples. The repeatability of the QC samples can reflect the stability of the instrument throughout the entire analysis process during data analysis. It also serves to identify variables with high variability in the analysis system, ensuring the reliability of the results.

### 2.10 Statistical analysis

Experimental data were analyzed using SPSS 22.0 (IBM, Chicago, IL, USA)., with one-way analysis of variance and Duncan’s test employed to determine any significant differences among the groups (p ≤ 0.05). Principal component analysis scores and loadings were used to evaluate the effects of the five treatments on the *A. ramosum* flowers.

## 3 Results

### 3.1 Different fertilizers promote the growth of *Allium ramosum* flowers

As seen in **Supplementary Figures S1A–D**, all fertilizer treatments enhanced the fresh weight, dry weight, inflorescence stem height, and corolla diameter of *A. ramosum* flowers as compared to the control (CK). Specifically, the B+S treatment was associated with notable 112, 223, and 29% increases in the fresh weight, dry weight, and corolla diameter, respectively, while B+A treatment significantly boosted the inflorescence stem height, with a 0.24-fold increase observed.

### 3.2 Promotion of the antioxidant capacity of *A. ramosum* flowers by different fertilizers

**Figure 1:**
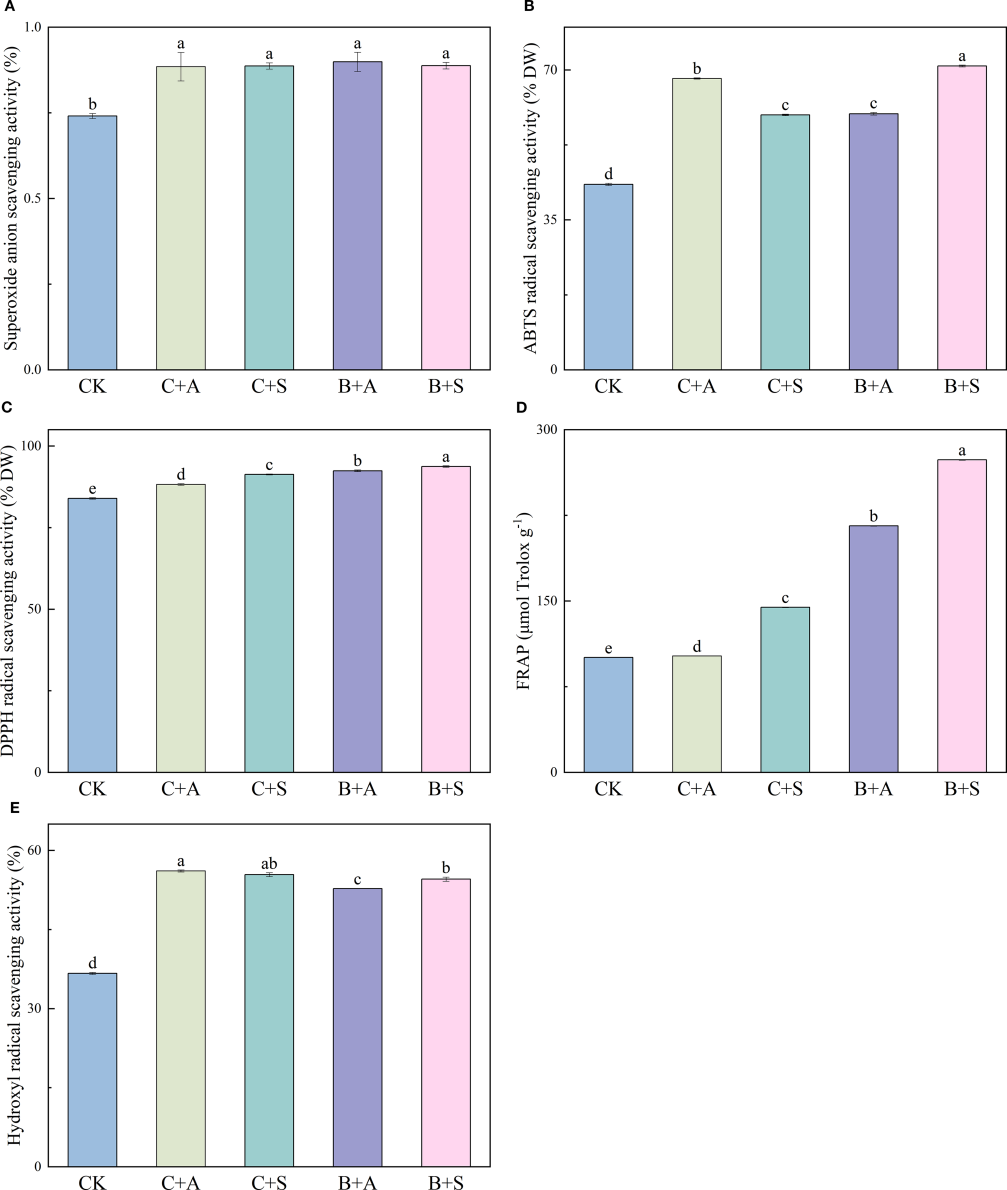
Illustrates the effects of different fertilizer treatments on the superoxide anion radical scavenging rate (SASR) **(A)**, 2,2’-Azino-bis(3-ethylbenzothiazoline-6-sulfonic acid) diammonium salt scavenging rate (ABTS) **(B)**, 2,2-diphenyl-1-picrylhydrazyl (DPPH) radical scavenging capacity **(C)**, ferric reducing antioxidant power (FRAP) **(D)**, and hydroxyl radical scavenging activity (HRSC) **(E)**. CK, C+A, C+S, B+A, and B+S represent the control group, chemical fertilizers combined with 0.2% amino acid fertilizer treatment group, chemical fertilizers combined with algae extract treatment group, compound microbial fertilizer combined with 0.2% amino acid fertilizer treatment group, and compound microbial fertilizer combined with 0.2% algae extract treatment group, respectively. Values are mean ± standard deviation of five replicates. Different lowercase letters indicate significant differences between treatments (p < 0.05).

Compared with the control (CK), all treatments, C+A, C+S, B+A, and B+S, were found to notably augment the superoxide anion radical scavenging rate (SASR), with 102, 103, 112, and 104% increases observed, respectively. (**Figure 1A**). C+A and B+S led to significant improvements in the ABTS scavenging rate as compared to CK, with increases of 57.1% and 63.8%, respectively (**Figure 1B**), while all treatments, C+A, C+S, B+A, and B+S, led to marked enhancements of the DPPH scavenging rates (5%, 9%, 10%, and 11.7%, respectively; **Figure 1C**); B+A and B+S increased the FRAP content 1.15- and 1.72-fold, respectively (**Figure 1D**); and C+A, C+S, B+A, and B+S significantly increased the HRSC by 53, 51.1, 43.9, and 48.7%, respectively (**Figure 1E**).

### 3.3 Promotion of the antioxidant enzyme activity of *A. ramosum* flowers by different fertilizers

In contrast to the control (CK), C + A, C + S, B + A, and B + S notably augmented the POD content, with increases of 9, 18.1, 28.2, and 55.8% observed, respectively (**Supplementary Figure S2A**); while C+A, C+S, B+A, and B+S significantly increased the PPO content by 44.8, 71.8, 48.8, and 50.4%, respectively (**Supplementary Figure S2B**); and C+A, C+S, B+A, and B+S significantly increased the SOD content, with increases of 32.5, 34.4, 55.8, and 36.2%, respectively (**Supplementary Figure S2C**).

### 3.4 Inhibition of the MDA and O2• − content in *A. ramosum* flowers under treatment with different fertilizers

Compared to the control (CK), the C+A, C+S, B+A, and B+S significantly reduced the MDA content by 22.4, 34.4, 29.6, and 47.2%, respectively (**Supplementary Figure S3A**), while C+A, C+S, B+A, and B+S significantly reduced the superoxide anion (O2• −) content by 9, 12, 15.5, and 29.2%, respectively (**Supplementary Figure S3B**).

### 3.5 Effects of different fertilizers on the TAA, SS, SP, and GSH contents in *A. ramosum* flowers

**Figure 2:**
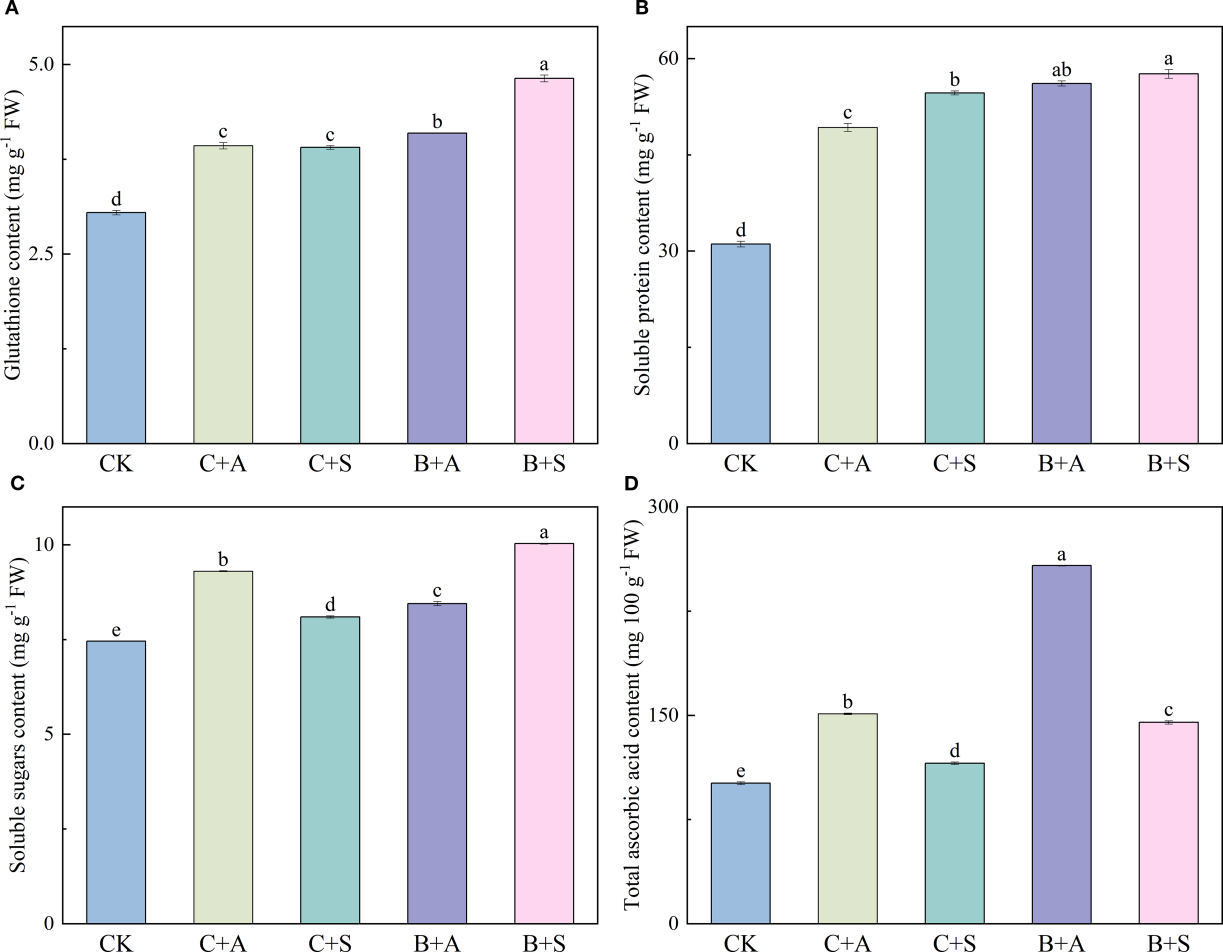
Depicts the effects of different fertilizer treatments on the content of Glutathione (GSH) **(A)**, Soluble protein content (SP) **(B)**, Soluble sugars content (SS) **(C)**, and Total ascorbic acid content (TAA) **(D)**. CK, C+A, C+S, B+A, and B+S represent the control group, chemical fertilizers combined with 0.2% amino acid fertilizer treatment group, chemical fertilizers combined with algae extract treatment group, compound microbial fertilizer combined with 0.2% amino acid fertilizer treatment group, and compound microbial fertilizer combined with 0.2% algae extract treatment group, respectively. Values are mean ± standard deviation of five replicates. Different lowercase letters indicate significant differences between treatments (p < 0.05).

The application of various fertilizers substantially influenced the levels of GSH, SS, SA, and TAA within the *A. ramosum* flowers. Compared to the control (CK), C+A, C+S, B+A, and B+S notably elevated the GSH content by 28.9, 28.2, 29.6, and 58.1%, respectively (**Figure 2A**); B+A and B+S increased the SP content 80 and 85%, respectively (**Figure 2B**); and C+A, C+S, B+A, and B+S led to a significant increase in the SS content, with enhancements of 24.8, 9, 13.3, and 34.6%, respectively (**Figure 2C**).

### 3.6 Effects of different fertilizers on the flavone, flavonoid, polyphenol, and total phenols contents in *A. ramosum* flowers

**Figure 3:**
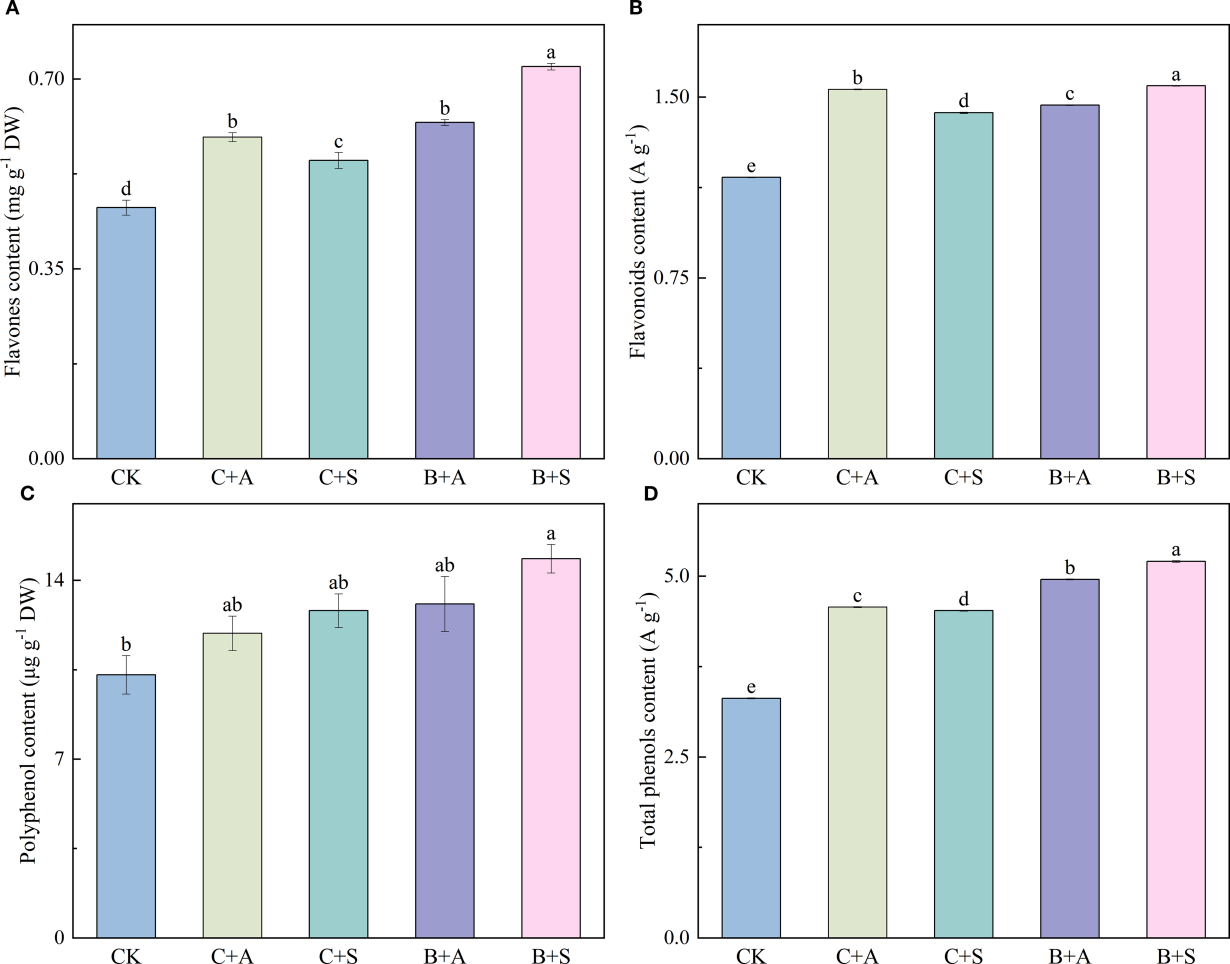
Illustrates the effects of different fertilizer treatments on the content of Flavones **(A)**, Flavonoids **(B)**, Polyphenol **(C)**, and Total Proline **(D)**. CK, C+A, C+S, B+A, and B+S represent the control group, chemical fertilizers combined with 0.2% amino acid fertilizer treatment group, chemical fertilizers combined with algae extract treatment group, compound microbial fertilizer combined with 0.2% amino acid fertilizer treatment group, and compound microbial fertilizer combined with 0.2% algae extract treatment group, respectively. Values are mean ± standard deviation of five replicates. Different lowercase letters indicate significant differences between treatments (p < 0.05).

The different fertilizers had significant impacts on the flavone, flavonoid, polyphenol, and proline contents in the *A. ramosum* flowers. Compared with the control (CK), C+A, C+S, B+A, and B+S significantly increased the flavone content by 28.1, 18.8, 33.9, and 56.1%, respectively (**Figure 3A**); C+A and B+S, significantly increased the flavonoid content by 31.3 and 32.6%, respectively (**Figure 3B**); B+S and B+A, significantly increased the polyphenol content by 44.2 and 27%, respectively (**Figure 3C**); and C+A, C+S, B+A, and B+S led to notable increases in the proline content, with enhancements of 38.1, 36.6, 49.7, and 57.1%, respectively (**Figure 3D**).

### 3.7 Effects of different fertilizers on the proline and free amino acid contents in *A. ramosum* flowers

The different fertilizers also had a significant impact on the proline and free amino acid contents of the *A. ramosum* flowers, with C+A, C+S, B+A, and B+S treatments associated with significant increases of 74.7, 67.4, 31, and 71.9% in the proline content, respectively (**Supplementary Figure S4A**); and C+A, C+S, B+A, and B+S significantly increasing the Free Amino Acid content by 46.7, 49.6, 41.1, and 29.6%, respectively (**Supplementary Figure S4B**), as compared to CK.

### 3.8 Correlation analysis

**Figure 4:**
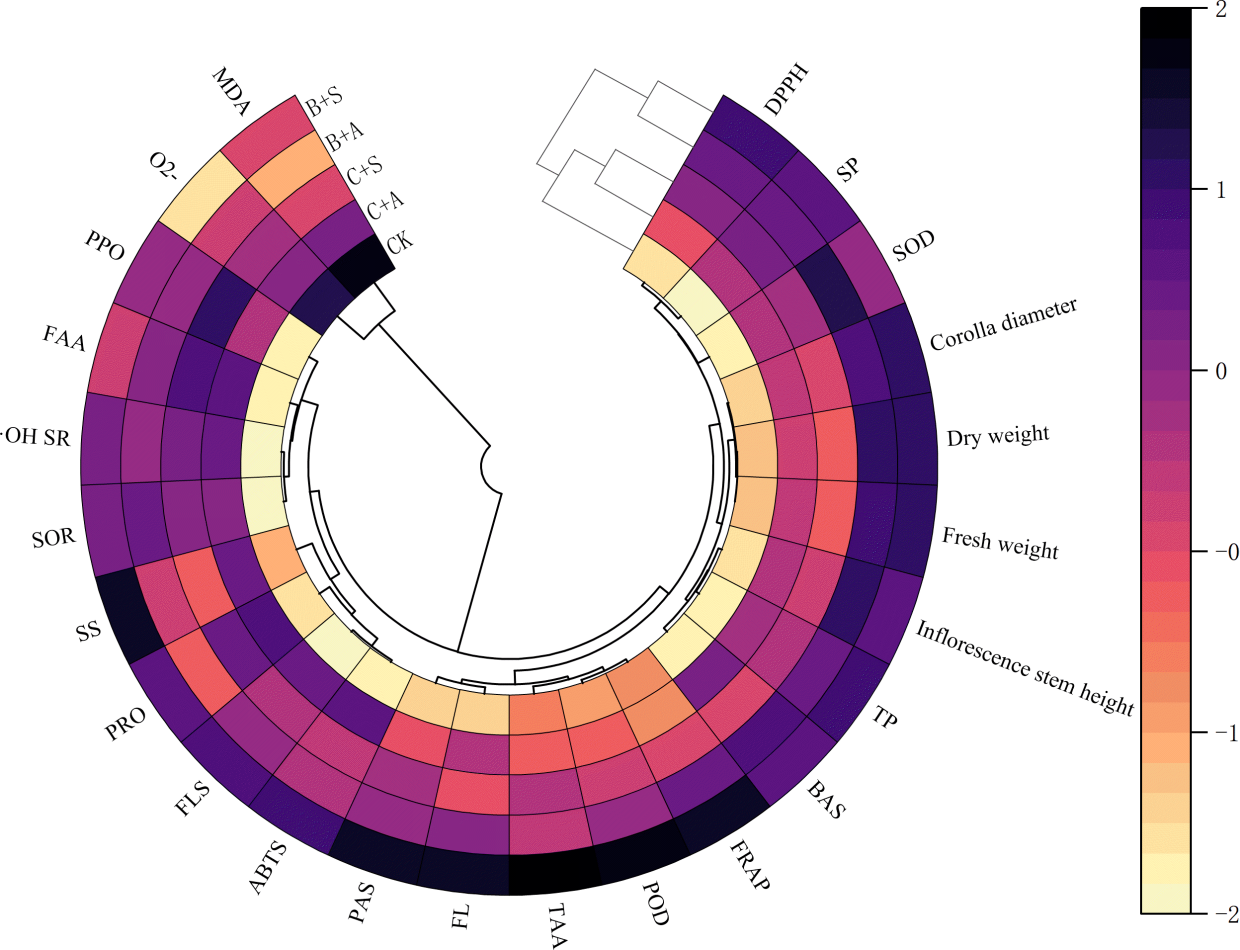
Presents a heatmap correlating different indices of *Allium ramosum* flowers with different fertilizer treatments. CK, C+A, C+S, B+A, and B+S represent the control group, chemical fertilizers combined with 0.2% amino acid fertilizer treatment group, chemical fertilizers combined with algae extract treatment group, compound microbial fertilizer combined with 0.2% amino acid fertilizer treatment group, and compound microbial fertilizer combined with 0.2% algae extract treatment group, respectively.

Pearson’s correlation analysis was used to test the correlations between different variables (**Figure 4**). The results indicated positive correlation between B+S and the SS content, flavonoid content, ABTS scavenging rate, proline content, polyphenol content, flavone content, POD, Corolla diameter, DW, FW, FRAP, and DPPH scavenging rate for *A. ramosum* flowers; with negative correlation observed for B+S and O2• − positive correlation observed between B+A and the TAA content, alkaloids, SOD, and Inflorescence stem height; negative correlation between B+A and MDA levels; significant positive correlation observed between C+S and both polyphenol oxidase activity and free amino acids content; significant positive correlation between C+A and proline content; and negatively correlation for C+A and FRAP.

**Figure 5:**
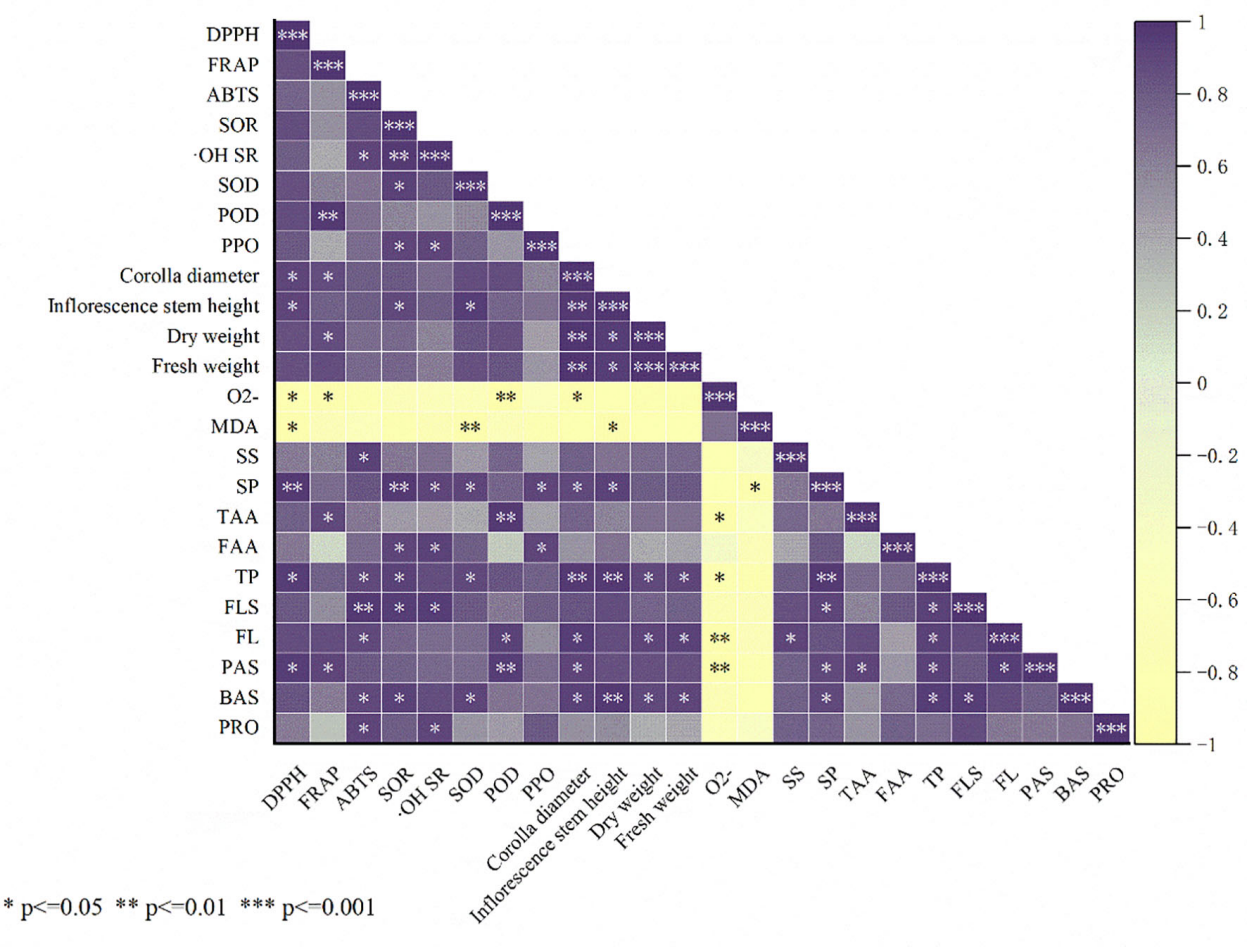
Shows the Pearson correlation coefficients between indices of *Allium ramosum* flowers. *, **, and *** represent significantly different correlations between treatment and control at p < 0.01, p < 0.05, and p < 0.001, respectively.

The Spearman’s rank correlation method was employed to examine the relationships between the various fertilizer applications and the growth metrics, antioxidant proficiency, and nutritional attributes of the *A. ramosum* flowers, with results indicating positive correlation between the DPPH scavenging rate and inflorescence stem height, corolla diameter, polyphenol content, proline content, and SP content and negative correlation with MDA and superoxide anions (O2• −). FRAP was positively correlated with corolla diameter, dry weight, polyphenol content, and POD, and negatively correlated with O2• − and the ABTS scavenging rate was positively correlated with proline, alkaloids, flavones, flavonoids, soluble sugars, and HRSC (**Figure 5**).

### 3.9 GC-MS analysis

**Figure 6:**
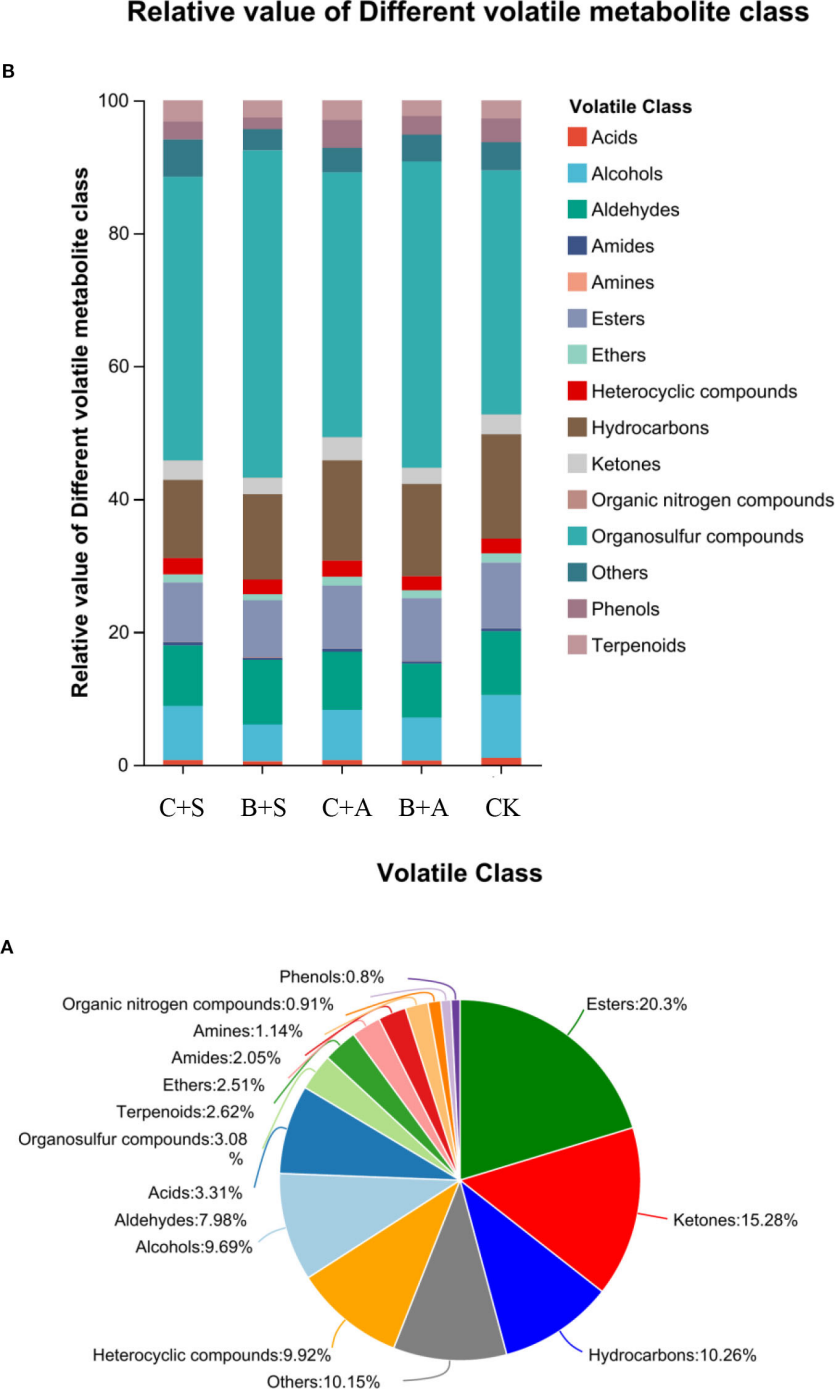
Volatile flavor components under different biostimulant treatments **(A)** Relative value of different volatile metabolite class **(B)** (CK, C+A, C+B, B+A, B+S).

Before and after the biostimulant treatments, a total of 504 volatile compounds were identified in the *Allium ramosum* flowers, including 23 Terpenoids, 10 Amines, 8 organic nitrogen compounds, 178 Esters, 29 Acids, 134 Ketones, 18 Amides, 85 Alcohols, 70 Aldehydes, 88 Others, 22 Ethers, 87 Heterocyclic compounds, 27 Organosulfur compounds, 90 Hydrocarbons, and 7 Phenols (**Figure 6A**).

**Figure 7:**
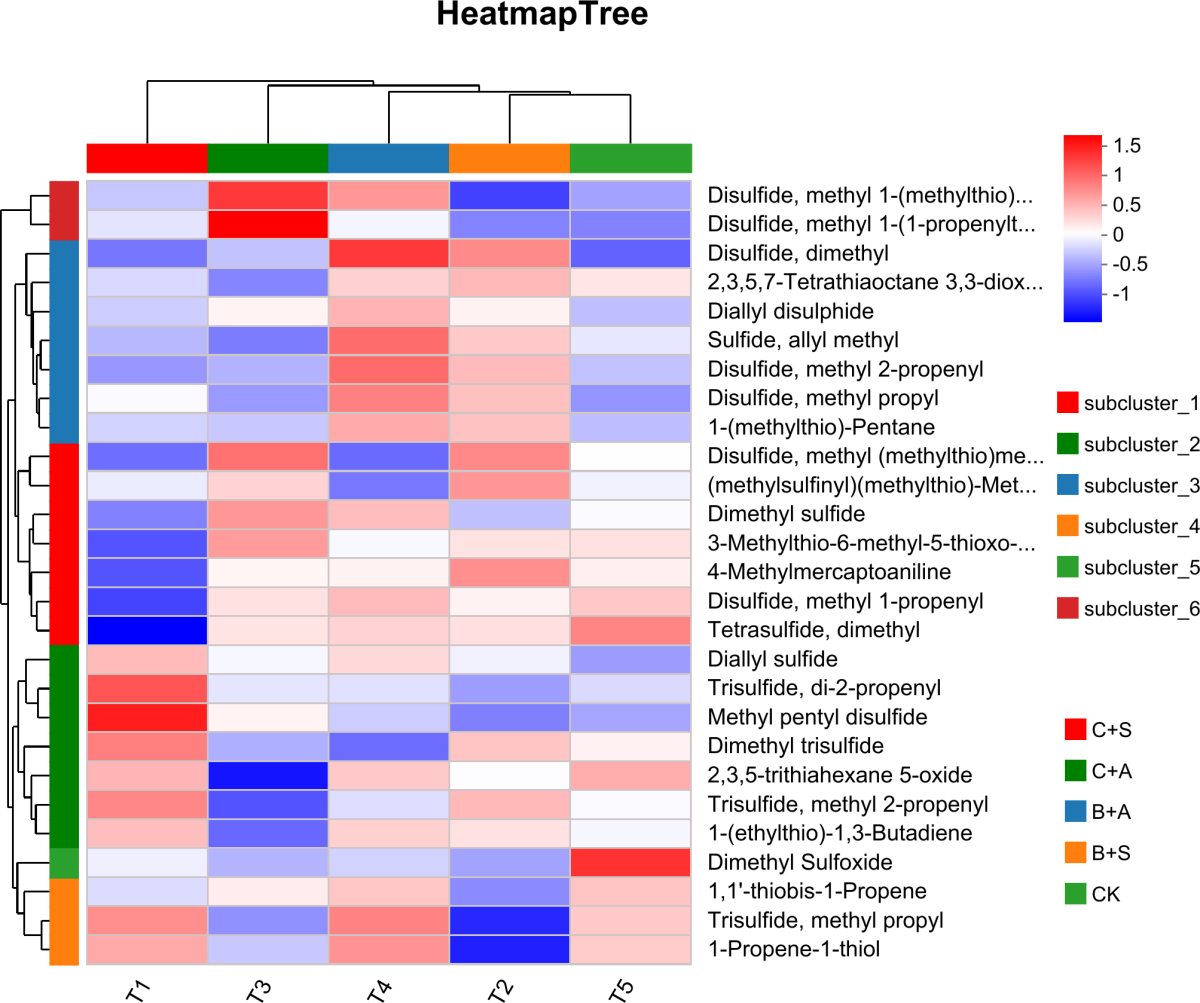
Thermal maps and hierarchical clustering of volatile compounds in *Allium ramosum* flowers before and after treatment with different biostimulants.

**Figure 8:**
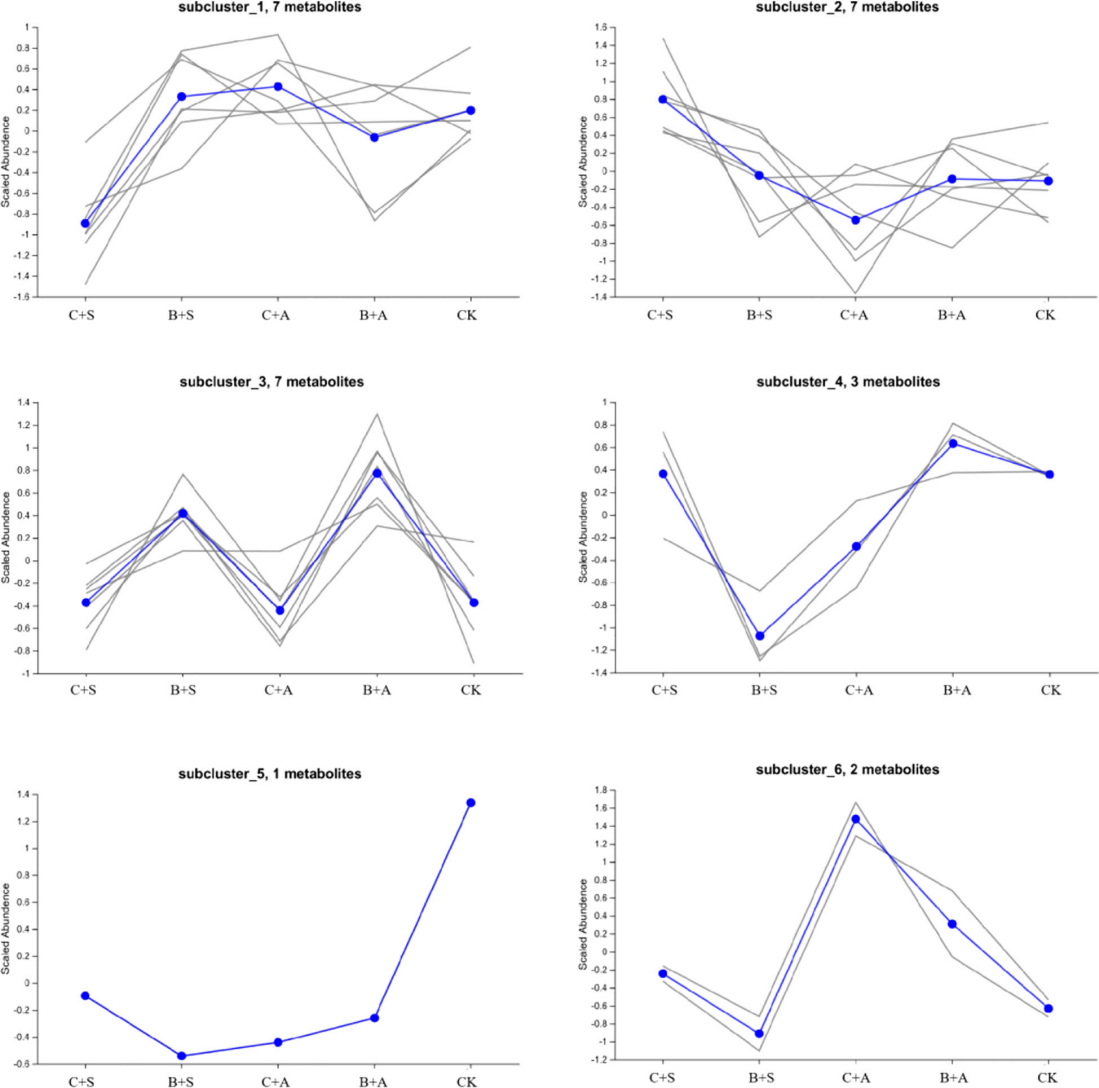
Trend chart of sub-clustering for the differential volatile sulfur compounds under different biostimulant treatments.

Compared to the CK, all treatments significantly increased the relative content of organic sulfur compounds. The B+S, B+A, C+S, and C+A treatments increased the content by 12.5%, 9.4%, 4.9%, and 3.1%, respectively, compared to CK (**Figure 6B**). The results indicate that the effects of B+S and B+A on the content of organic sulfur compounds are greater than those of C+S and C+A (**Figure 7**). To visually present the trend of metabolite changes under biostimulant treatments, a clustering analysis was performed on the standardized differential metabolite content. When *Allium ramosum* flowers were treated with B+A, the relative expression levels of Disulfide, methyl 1-(methylthio)propyl, Disulfide, dimethyl, 2,3,5,7-Tetrathiaoctane 3,3-dioxide, Diallyl disulphide, Sulfide, allyl methyl, Disulfide, methyl 2-propenyl, Disulfide, methyl propyl, 1-(methylthio)-Pentane, Dimethyl sulfide, Disulfide, methyl 1-propenyl, Tetrasulfide, dimethyl, Diallyl sulfide, 2,3,5-trithiahexane 5-oxide, 1-(ethylthio)-1,3-Butadiene, 1,1’-thiobis-1-Propene, Trisulfide, methyl propyl, and 1-Propene-1-thiol were high. When *Allium ramosum* flowers were treated with B+S, the relative expression levels of Disulfide, dimethyl, 2,3,5,7-Tetrathiaoctane 3,3-dioxide, Sulfide, allyl methyl, Disulfide, methyl 2-propenyl, Disulfide, methyl propyl, 1-(methylthio)-Pentane, Disulfide, methyl (methylthio)methyl, (methylsulfinyl)(methylthio)-Methane, 3-Methylthio-6-methyl-5-thioxo-2,5-dihydro-1,2,4-triazine, 4-Methylmercaptoaniline, Tetrasulfide, dimethyl, Dimethyl trisulfide, Trisulfide, methyl 2-propenyl, and 1-(ethylthio)-1,3-Butadiene were high (**Figure 7**). These 27 different organosulfur compounds could be classified into 6 subcategories based on their relative content. An overall upward trend was observed in the third subcategory, where the content of these 8 metabolites in the B+S and B+A samples was higher than that in the C+S, C+A, and CK samples, indicating that 7 of these metabolites (Diallyl disulphide, Disulfide, dimethyl, Sulfide, allyl methyl, Disulfide, methyl propyl, Disulfide, methyl 2-propenyl, 1-(methylthio)-Pentane, and 2,3,5,7-Tetrathiaoctane 3,3-dioxide) are associated with the B+S and B+A samples (**Figure 8**).

## 4 Discussion

### 4.1 Effect of different fertilizers on flower growth in *A. ramosum*

Numerous studies have demonstrated that the application of external amino acid-based fertilizers or substances derived from algal extracts markedly enhances plant growth and development.

Within the context of this investigation, a notable enhancement in the corolla diameter was observed under all four treatments, along with dry mass, fresh mass, and the inflorescence stem length of the *A. ramosum* blossoms as compared to CK. Notably, B+A and B+S yielded significantly better outcomes than C+A and C+S (**Supplementary Figures S1A-D**), suggesting that using a compound microbial fertilizer as a base with amino acid or algal extract as a foliar fertilizer can effectively promote the growth of *A. ramosum* flowers. Echoing the conclusions of Khan et al. (2019), the current study confirmed that using amino acid-based fertilizers promotes plant growth and increases biomass (Khan et al., 2019). In parallel, a liquid amino acid fertilizer derived from pig bristles has also been found to augment crop yield (Wang et al., 2019). These results suggest that amino acids stimulate plant growth and augments agricultural yield. In the present study, the application of amino acid fertilizers resulted in an increase in the fresh and dry biomass of *A. ramosum*, which is consistent with earlier findings by Khan et al. (2019) and Wang et al. (2019) suggesting that amino acid fertilizers may enhance the growth of *A. ramosum* flowers by stimulating the endogenous synthesis of hormones, facilitating improved nutrient uptake. Amino acids have also been found able to activate specific transporters, facilitating the ability of plants to more effectively absorb and utilize nutrients (Sharma and Dietz, 2006). The promotion of inflorescence stem height observed for B+A was superior to that by C+A, possibly because of the interaction of microorganisms in the compound microbial fertilizer with the minerals and organic substances in the soil promoting the absorption of nutritional elements by the plants.

Previous research has indicated that both algal extracts and their isolated constituents induce pronounced physiological reactions in plants, resulting in enhanced stem elongation and increased root biomass (Yakhin et al., 2017; Hernández-Herrera et al., 2014). Microalgal extracts, particularly those containing polysaccharides, have been shown to foster the growth of agricultural crops (Ronga et al., 2019; Garcia-Gonzalez and Sommerfeld, 2016). Sugars function as both potent signaling agents and direct precursors for intermediary metabolism, thereby facilitating plant growth and development (Zheng et al., 2016). The presence of macro- and micronutrients, vitamins, and phytohormones in algal extracts may also contribute to this process (Garcia-Gonzalez and Sommerfeld, 2016; Ghaderiardakani et al., 2019). For example, studies have indicated that the potassium in seaweed extracts can positively modulate the water relations of the treated plants, enhance the photosynthetic activity, and promote meristematic growth (Hernández-Herrera et al., 2014). The superior promotion of corolla diameter, DW, and FW for *A. ramosum* flowers as a result of B+S application as compared to C+S is possibly because the combination of a variety of microorganisms in the compound microbial fertilizer with the active components in the algal extract enhances the biological activities of both. The growth hormones and trace elements in algal extracts can promote the growth and metabolism of microorganisms, which can further decompose and transform the nutrients in the algal extract, rendering them more easily absorbed by plants.

### 4.2 Fertilizer effects on *A. ramosum* flower antioxidant capacity

*Allium* plants are consumed globally as food, and their impact on human health is significant because of their rich content of bioactive compounds, with antioxidant, anti-inflammatory, and anticancer activities (Yang et al., 2023). In a study conducted by Pourzand et al. (2016), the authors reported the consumption of certain *Allium* plants in association with a reduced risk and incidence of breast cancer. The abundance of phytochemicals in *Allium* vegetables has meant that their health benefits have been fully explored, holding promise for the development of new foods and nutritional supplements.

Our findings indicate significantly higher FRAP, 2,2’-Azino-bis(3-ethylbenzothiazoline-6-sulfonic acid) diammonium salt

scavenging rate, DPPH scavenging rate, superoxide anion radical scavenging rate, and HRSC for *A. ramosum* flowers treated with C+A, C+S, B+A, and B+S as compared to CK (**Figures 1A–E**). Notably, the SASR was significantly higher for the B+A treatment than the other treatments, whereas the FRAP, ABTS scavenging rate, DPPH scavenging rate, and HRSC were significantly higher for the B+S treatment (**Figures 1A–E**).

The antioxidant properties of garlic are closely associated with its phenolic composition. Kumar and Kumar (2023) found that elevated concentrations of polyphenols led to enhanced antioxidant efficacy in various plant tissues in the *Allium* species, including the leaves, roots, bulbs, and pericarps. Concurrently, Mollica et al. (2018) revealed that the phenolic constituents of *Allium* scoroprasum L. floral extracts are abundant, conferring potent antioxidant capabilities and functioning as effective metal chelators and free radical scavengers (as evidenced by the DPPH and ABTS scavenging rates). Dai et al. (2023) highlighted the pivotal role of phenolic compounds and ascorbic acid in the antioxidant mechanism of leeks. Flavonoids, which are prevalent across a wide array of plant species, are integral to the antioxidant, anticancer, antimicrobial, and antimutagenic defenses of a plant and constitute a significant class of antioxidants synthesized by plants for their survival (Treutter, 2006; Ghasemzadeh et al., 2010; Sharma et al., 2014).

Correlation analysis indicated that proline content is significantly positively correlated with the DPPH, ABTS, and the superoxide anion radical scavenging rates, whereas polyphenol content is significantly positively correlated with the DPPH and ABTS scavenging rates (**Figure 5**). These results suggest that an increase in the proline and flavonoid content, among other bioactive substances, may be the reason for the enhanced scavenging and reduction capacity of *A. ramosum* flowers (**Figures 3A–D**). The antioxidant activity (DPPH scavenging rate, ABTS scavenging rate, FRAP, superoxide anion radical scavenging rate, and HRSC) was significantly correlated with proline, polyphenol, flavonoid, flavone, and TAA contents (**Figures 4**, **7**). B+A treatment yielded analogous outcomes. This finding provides additional evidence indicating that the enhanced antioxidant capacity of *A. ramosum* blossoms is likely associated with the augmentation of the bioactive compound content. Interestingly, the DPPH and ABTS scavenging rates, FRAP, superoxide anion radical scavenging rate, and HRSC treatments were significantly higher following B+A and B+S treatment, indicating the possibility of a synergistic effect between compound microbial fertilizer and tri-amino acid fertilizer or algal extracts.

### 4.3 Fertilizer effects on *A. ramosum* flower antioxidant enzyme activity

MDA serves as an indicator for the extent of impairment in plant cell membranes (Du et al., 2024). The progressive generation of toxic oxygen species culminates in lipid peroxidation and subsequent deterioration of plant cell membrane integrity, hastening cellular apoptosis (Raju et al., 2020). ROS are noxious by-products of metabolism, in addition to an association with oxidative and nitrosative stressors, all of which exert detrimental effects on plant physiology (Samanta et al., 2024). In the present study, all treatments reduced the levels of MDA and O2• − (**Supplementary Figures S3A, B**) while significantly increasing the POD and SOD contents (**Supplementary Figures S2A, B**). These results align with the conclusions of Al-Karaki and Othman (2023), wherein the utilization of amino acid fertilizers was observed to enhance antioxidant activity in lettuce. However, the results are different from those reported by Zhang et al. (2022), who suggested a non-significant effect for amino acid fertilizers on the SOD content in Trollius chinensis, suggesting a need for additional investigation. However, Ali et al. (2023) observed an increase in POD activity, polyphenol content, and proline content following the application of algal extracts via foliar spray in tomato and sweet pepper plants, and Orhan et al. (2004) highlighted the fact that SOD and POD are principal cellular enzymes that safeguard plants against ROS-induced damage, a notion that concurs with our findings as indicated by correlation analysis (**Figures 4**, **5**). Treatment with B + A and B + S had a significant effect on the POD and SOD contents in *A. ramosum* flowers, with MDA negatively correlated with POD and O2• − production negatively correlated with SOD.

### 4.4 Fertilizer effects on *A. ramosum* flower quality

The nutritional attributes of vegetables are instrumental in safeguarding their quality and guaranteeing their nutritional interity (Kathi et al., 2024; Wang et al., 2021). The data obtained in our investigation revealed that all studied treatments led to a marked enhancement in the concentrations of GSH, TAA, soluble sugars, SP, proline, polyphenols, flavones, and flavonoids in leeks as compared with CK (**Figures 2A–D**, **3A–D**). GSH, an important non-enzymatic antioxidant, plays a crucial role in plants by clearing ROS and regulating the cellular redox balance. Studies have shown that GSH can directly react with ROS (O2• −) to form complexes, thereby reducing the oxidative damage (Hasanuzzaman et al., 2017). In addition, GSH can maintain the reduced state of other antioxidants (such as vitamins C and E) and indirectly protect cell membranes (Foyer and Noctor, 2005). These studies indicate that GSH plays a crucial role in plant growth and development (E et al., 2024). Numerous investigations have revealed that the incorporation of amino acid-derived biostimulants can exert a favorable influence on the proline content, flavone content, and antioxidant potential (Parađiković et al., 2011; Pourzand et al., 2016; Shafie et al., 2021). For instance, the administration of diverse biostimulants, such as algal extracts and amino acids, and protein hydrolysates, has been documented to augment the proline content and total flavonoid content and antioxidant capacity in hydroponically cultivated sweet pepper fruits (Parađiković et al., 2011), Achillea leaves (Shafie et al., 2021), tomato fruits (Caruso et al., 2019), and lettuce leaves (Zhou et al., 2022).

The results of these studies suggest that the application of biostimulants is a good strategy for increasing the yield of nutritious vegetable crops with lower environmental impact (Parađiković et al., 2011; Shafie et al., 2021). It is noteworthy that GSH, TAA, soluble sugar, SP, proline, polyphenol, flavone, and flavonoid contents were significantly increased under B+A and B+S-treatment (**Figures 3A–D**, **4A–D**, **4**, **5**). According to previous studies, the accumulation of proline in plants aids in maintaining cellular osmotic equilibrium, which in turn enables plants to withstand various environmental challenges, such as drought, salt-induced stress, and cold temperatures (Wang et al., 2020). Proline has been found to alleviate oxidative stress in plants by modulating the activity and expression of antioxidant enzymes and acting as a scavenger for ROS (Sehar et al., 2021). For example, proline accumulation can enhance the antioxidant defense systems in wheat seeds (Sehar et al., 2021) and stabilize protein structures (Szabados and Savouré, 2010). These studies indicate that proline plays a multifaceted role in plant growth. However, proline accumulation is not isolated, and is closely related to the metabolism of free amino acids content. Free amino acids content are important nitrogen sources in plants and participate in various physiological processes, such as protein and hormone synthesis.

The types and content of free amino acids content differ in different plants under various conditions (Sun et al., 2017; Moore et al., 2022). Studies have shown the types and contents of free amino acids in plants can undergo significant changes to adapt to new physiological requirements when a plant is placed under adverse stress (Hanif et al., 2021). These changes can affect the growth, development, and antioxidant capacity of plants. Furthermore, free amino acids serve as substrates for antioxidants and participate in ROS scavenging to alleviate oxidative damage (Choińska et al., 2022).

The results of our study demonstrate that the proline and free amino acid contents were markedly elevated in leek plants subjected to all treatments as compared to the control (**Supplementary Figures S4A, B**). In particular, a substantial increase was observed in the proline and free amino acid content for the B + A and B + S treatment groups as compared to the C+A or C+S treatment groups (**Supplementary Figures S4A, B**; **Figures 4**, **5**).

### 4.5 The effects of different biostimulants on the volatile sulfur compounds in *Allium ramosum* flowers

Among the seven metabolites in the third subclass, Diallyl Disulphide, Disulfide, methyl 2-propenyl, and Sulfide, allyl methyl are metabolites associated with oxidation during the aging process. Studies have shown that Diallyl Disulphide can induce the expression of antioxidant enzymes by regulating the Nrf2-ARE signaling pathway, thereby protecting cells from oxidative stress. This suggests that Diallyl Disulphide may exert an indirect antioxidant effect by regulating the antioxidant defense mechanism in plants (Shang et al., 2019). Disulfide, methyl 2-propenyl is a natural sulfur-containing compound in garlic with antioxidant activity. Like other sulfur-containing compounds, Disulfide, methyl 2-propenyl can scavenge ROS, protecting cells from oxidative damage (Vega-Hissi et al., 2019). Therefore, we speculate that Diallyl Disulphide, Disulfide, methyl 2-propenyl, and Sulfide, allyl methyl are related to the increased 2,2’-Azino-bis(3-ethylbenzothiazoline-6-sulfonic acid) diammonium salt (ABTS) scavenging rate, DPPH scavenging rate, and FRAP content, as well as the decreased MDA content and O2• − content in the B+S and B+A samples.

## 5 Conclusion

This study aims to investigate the effects of four fertilization methods on the growth, volatile compounds, and antioxidant capacity of *Allium ramosum* flowers: conventional fertilizer + 0.2% amino acid fertilizer (C+A), conventional fertilizer + 0.2% algal extract (C+S), 225.00 kg/ha compound microbial fertilizer + 0.2% amino acid fertilizer (B+A), and 225.00 kg/ha compound microbial fertilizer + 0.2% algal extract (B+S). The main conclusions are as follows: First, B+A and B+S significantly promote the growth of *Allium ramosum* flowers. Second, they respectively significantly increase the soluble protein content, soluble sugar content, total ascorbic acid content, proline content, polyphenol content, flavonoid content, and flavone content in *Allium ramosum* flowers, improving the quality and antioxidant activity of the flowers, thereby enhancing their commercial value. Third, they effectively increase the content of sulfides and aromatic volatile components, improving the characteristic flavor compounds of *Allium ramosum* flowers. In summary, treatments B+A and B+S are the best choices for promoting the growth of *Allium ramosum* flowers.

## Data Availability

The raw data supporting the conclusions of this article will be made available by the authors, without undue reservation.
