# Supplementary material for: Impact of different plant growth promoters on the physicochemical properties, volatile compounds, and antioxidant capacity of Allium ramosum flowers
Source: Front Plant Sci. 2025 Oct 6;16:1606438. doi: 10.3389/fpls.2025.1606438 (PMC12536015; doi:10.3389/fpls.2025.1606438)
Supplement: Supplementary file 1 [file DataSheet1.docx]

**Fig. S1** Shows the effects of different fertilizer treatments on fresh *Allium ramosum* flower Fresh weight (A), flower Dry weight (B), Inflorescence stem height (C), and Corolla diameter (D). CK, C+A, C+S, B+A, and B+S represent control group, chemical fertilizers combined with 0.2% amino acid fertilizer treatment group, chemical fertilizers combined with algae extract treatment group, compound microbial fertilizer combined with 0.2% amino acid fertilizer treatment group, and compound microbial fertilizer combined with 0.2% algae extract treatment group, respectively. Values are mean ± standard deviation of five replicates. Different lowercase letters indicate significant differences between treatments (p < 0.05).

**Fig. S2** Demonstrates the effects of different fertilizer treatments on the activities of peroxidase (POD) (A), polyphenol oxidase (PPO) (B), and superoxide dismutase (SOD) (C). CK, C+A, C+S, B+A, and B+S represent the control group, chemical fertilizers combined with 0.2% amino acid fertilizer treatment group, chemical fertilizers combined with algae extract treatment group, compound microbial fertilizer combined with 0.2% amino acid fertilizer treatment group, and compound microbial fertilizer combined with 0.2% algae extract treatment group, respectively. Values are mean ± standard deviation of five replicates. Different lowercase letters indicate significant differences between treatments (p < 0.05).





**Fig. S3** Illustrates the effects of different fertilizer treatments on the levels of Superoxide anion (O_2_^⋅−^) (A) and Malondialdehyde (MDA) (B). CK, C+A, C+S, B+A, and B+S represent the control group, chemical fertilizers combined with 0.2% amino acid fertilizer treatment group, chemical fertilizers combined with algae extract treatment group, compound microbial fertilizer combined with 0.2% amino acid fertilizer treatment group, and compound microbial fertilizer combined with 0.2% algae extract treatment group, respectively. Values are mean ± standard deviation of five replicates. Different lowercase letters indicate significant differences between treatments (p < 0.05).





**Fig. S4** Demonstrates the effects of different fertilizer treatments on the content of Proline (A) and Free amino acid (B). CK, C+A, C+S, B+A, and B+S represent the control group, chemical fertilizers combined with 0.2% amino acid fertilizer treatment group, chemical fertilizers combined with algae extract treatment group, compound microbial fertilizer combined with 0.2% amino acid fertilizer treatment group, and compound microbial fertilizer combined with 0.2% algae extract treatment group, respectively. Values are mean ± standard deviation of five replicates. Different lowercase letters indicate significant differences between treatments (p < 0.05).
